# Mechanical Dentistry—Past and Present

**Published:** 1869-01

**Authors:** J. P. H. Brown

**Affiliations:** Augusta, Georgia


DENTAL REGISTER.

Vol. XXIII.] JANUARY, 1869. [No. 1.

### Original Communications.

### MECHANICAL DENTISTRY—PAST AND PRESENT.

BY J. P. H. BROWN, AUGUSTA, GEORGIA.

Mechanical Dentistry includes more than mere manual dexterity in the use of tools. Like sculpture and painting, it gives the creations of expression and intelligence. No block of marble in the hands of the sculptor was ever more subject to his control than are the varied expressions of the mouth and its surroundings subject to the controlling power of the Dental Art.

The Dentist is not only an artisan, but *he must be an artist.* He can work by no set of rules, like the handicraftsman, but by the power of his art he has to infuse into his work a life—an expression—a soul. He can not be a mere copyist, because he has not always the natural teeth to copy from. His subject often comes toothless, with features contracted, and all natural expression of the mouth gone. In such cases he has to depend upon his æsthetic conceptions and knowledge of the physiognomical poles of the sentiments and emotions, and the harmony and blending of shades, forms, etc. Besides all this he should have a thorough acquaintance with anatomy, physiology, chemistry, metallurgy and mineralogy. Hence, the greater the acquirements and skill of the Dentist, and the more closely he *imitates* nature, the more perfectly will his artificial denture supply the place of the one lost.

Since mechanical Dentistry is so pre-eminently artistic, and demands, in order to accomplish the best results, a degree of skill second to no other profession, it may not be amiss to examine into its present "status." But here the questions naturally arise: Has it, during the last ten years, been progressing upward toward the æsthetic—the ideal of nature —or has it been downward toward simple mechanical routine? Has the tendency of the dental mind, during these years, been toward the discovery of a material for a base, or a method of mounting artificial teeth, producing the most perfect results, and, of course, requiring a corresponding degree of skill; or has the study been to find some *common*, *cheap* substance demanding neither skill, ability nor brains to work, by which a full set of "double-back-action" grinders, "warranted equal to nature," can be inserted for twenty dollars, or less? These questions to the Dental bungler may be of no moment, but to the practitioner whose aspirations are upward and onward, and who desires to see and to aid in placing his profession upon the same basis, and characterized by the same *esprit de corps*, as medicine or law, they afford material for thought—sad thought.

The circulars and advertisements of the venders of these *base* bases have become as common as those of the patent medicine quacks. They hold out the shabby inducement to use their stuff, that a few hours study, or a week, at farthest, is sufficient to master the manipulations of their wonderful invention; that there can be no misfits; and that the cost of material for a full set amounts to *only* the paltry sum of three shillings. Men who have neither the natural ability, nor the acquired qualifications to construct a passable gold job, to say nothing of continuous gum and block-work, grab at such a "trade," so easily learned, like a hungry spider at a fly. Such characters and their abettors are nothing more than machines, grinding out caoutchouc, or something else belonging to the same category, *indiscriminately* to all who are so unfortunate as to apply for a set of "masticators." The effect of all this is to drive talent from the laboratory in disgust. To call Dentistry a profession, under such circumstances, is preposterous. What gives dignity and standing to a profession? Unquestionably the long study, the close application, and the talent and skill demanded in order to practice it with success. If we wish to improve the professional "status" of mechanical Dentistry, we must labor to construct a class of work vieing nature as closely as possible; and such work can only be produced by the highest order of skill. Men may get *above* the professional level, but none should be far below it.

I will take the ordinary dental rubber for illustration. Now the great mass of rubber-workers use it indiscriminately, and make their patients believe that it is better than all other styles of work. They do not recommend it because they honestly believe it better, but because there is "more money in it," and they have no occasion to exercise much brain in putting it up. Rubber work, as usually constructed, with the moulded blocks and the stiffly and mechanically arranged teeth, often reminding one of a mouth filled with little pegs, may seemingly be very nice to a Dentist of low conceptions; but when placed by the side of a beautiful piece of continuous gum-work properly constructed, (and when thus made it is strong, durable, artistic, and the most cleanly of any) it is like comparing the common plaster of Paris images with the creations of an Angelo or a Powers.

The indiscriminate use of any style of work is prejudicial to the highest attainments. Hobbyism in mechanical Dentistry is just as short-sighted, and as productive of bad results as it is in the operative department. The rule should be to recommend *the very best* of which the case will admit.

But it is urged that cheap materials are required to "meet the wants of the poor," and other cases arising frequently in a full practice. Then confine their use to these "other cases" and to these "wants;" but even with the poor it is better to give them the best and let the profits be paid by the rich.

The introduction of cheap work, besides lowering the grade of qualifications for the laboratory, whereby an army of incompetents have been turned loose to prey upon the gullible public, has had the effect to cause less attention to be paid to the preservation of the natural teeth. The common mass let their teeth go to decay and destruction, when they can get a full denture of artificial ones (warranted of course) for less than they would have to pay a first-class operator to insert a gold filling. An esteemed Dental friend, of acknowledged skill and ability, and who has a large practice, when writing me a few weeks ago, made use of the following language; "My practice is more and more becoming one of extraction and the making of artificial teeth. This is a lamentable fact; but so it is, in spite of my twenty years hard labor to awaken the people to a full sense of the great value of operations upon the natural teeth." This, alas, is the experience of many.

Having shown that these cheap and common materials for a tooth-base, have not only impaired the respectability of the profession, but have really degraded the mechanical department to a "tinker's trade," (besides, done the people an irreparable injury) let us examine a little into the history of their introduction.

In the first place, Dentistry is unquestionably as much a part of medicine as surgery. It is so considered by our first physicians and surgeons, and so proclaimed by our Dental College professors. Dentistry being a part of medicine, it necessarily follows that the same rules and etiquette which are laid down to govern the professional conduct of the practitioners of medicine and surgery, should also govern the practitioners of Dental science.

As the medical profession owes its power, influence and skill to the perfectly free and unrestrained interchange of ideas and opinions, it is held to be derogatory for one of its members to patent any remedy, appliance, or principle of its application. As each member draws from the common fund of knowledge—the accumulated discoveries of centuries—he is morally and professionally bound to contribute his portion, either small or large, to the common stock. To infringe this law of the professional code is considered so great a misdemeanor, that the offender is no longer considered worthy the respect and confidence of the profession. But how different is this from the present ethical code of the Dental profession.

As soon as a member imagines he has made a discovery—without even waiting to test it—he hurries off to Washington to get it patented. It is true the rubber patents were taken out by men not members of the profession. Many of these patented so-called improvements are mere clap-traps—not worth a six-pence. Some are only old processes, slightly modified, that have long since died—been buried in disgust—now resurrected.

But, says the patent monger, he has a *legal* right to a patent. He must be paid for his long years of study, and for the money (mostly spent for his patent) expended in perfecting his invention. That "the laborer is worthy of his hire." I admit that all these rights are his—*the same* that are enjoyed by any mechanic—but deny that he has any right to claim the honors and privileges of a profession that owes its life to a free and liberal exchange of ideas and experience. It is time the various Dental Associations were awaking to a full sense of the mischief done by these excresences on Dental progress.

This jumping at every new fangled thing that comes up is not always, by any means, a correct way to test true progress. Great improvements are not developed by fits and starts. They are the result of time and of united energies. The history of the introduction and improvements in the manufacture of porcelain single teeth, carved blocks, continuous gum work, (Allen's improved) etc., show that from the time of the first conception of mineral teeth by Fauchard down to the improvement in their manufacture by Delabarre, and from the time of the latter down to the present beautiful productions, it has been idea added to idea, experience piled upon experience.

Mechanical Dentistry can only be kept from degenerating to the level of a common trade by avoiding cheap work; by using such materials and modes of construction upon which the highest skill can be placed; by practitioners ceasing to receive pupils, except such as have the requisite talents and education; and by a strict adherence to the code of professional ethics.

